# Objectively Measured Physical Activity Is Associated with Brain Volumetric Measurements in Multiple Sclerosis

**DOI:** 10.1155/2015/482536

**Published:** 2015-06-04

**Authors:** Rachel E. Klaren, Elizabeth A. Hubbard, Robert W. Motl, Lara A. Pilutti, Nathan C. Wetter, Bradley P. Sutton

**Affiliations:** ^1^Department of Kinesiology and Community Health, University of Illinois at Urbana-Champaign, Urbana, IL 61801, USA; ^2^Department of Bioengineering, University of Illinois at Urbana-Champaign, Urbana, IL 61801, USA

## Abstract

*Background*. Little is known about physical activity and its association with volumes of whole brain gray matter and white matter and deep gray matter structures in persons with multiple sclerosis (MS).* Purpose*. This study examined the association between levels of physical activity and brain volumetric measures from magnetic resonance imaging (MRI) in MS. *Method*. 39 persons with MS wore an accelerometer for a 7-day period and underwent a brain MRI. Normalized GM volume (NGMV), normalized WM volume (NWMV), and deep GM structures were calculated from 3D T1-weighted structural brain images. We conducted partial correlations (*pr*) controlling for demographic and clinical variables.* Results*. Moderate-to-vigorous physical activity (MVPA) was significantly associated with NGMV (*pr* = 0.370, *p* < 0.05), NWMV (*pr* = 0.433, *p* < 0.01), hippocampus (*pr* = 0.499, *p* < 0.01), thalamus (*pr* = 0.380, *p* < 0.05), caudate (*pr* = 0.539, *p* < 0.01), putamen (*pr* = 0.369, *p* < 0.05), and pallidum (*pr* = 0.498, *p* < 0.01) volumes, when controlling for sex, age, clinical course of MS, and Expanded Disability Status Scale score. There were no associations between sedentary and light physical activity with MRI outcomes.* Conclusion*. Our results provide the first evidence that MVPA is associated with volumes of whole brain GM and WM and deep GM structures that are involved in motor and cognitive functions in MS.

## 1. Introduction

The deterioration of whole brain gray matter (GM) and white matter (WM) volumes and volumes of deep GM structures (e.g., hippocampus, thalamus, caudate, putamen, and pallidum) occurs in people with multiple sclerosis (MS) [1, 2]. Such deterioration is associated with disability status [3] and cognitive dysfunction [4–6] in MS. For example, one study reported that whole brain GM volume correlated with disability status and cognitive impairment, as measured by the Expanded Disability Status Scale (EDSS) and Paced Auditory Serial Addition Test (PASAT), respectively, in a sample of 977 patients with MS [7].

We are unaware of research examining physical activity and its association with volumes of whole brain GM and WM and deep GM structures in people with MS. Nevertheless, there is consistent evidence for low levels of physical activity in MS, such that only 20% of persons with MS are meeting public health guidelines for moderate-to-vigorous physical activity (MVPA) [8, 9]. This is a concern, since studies have demonstrated that physical activity is associated with brain volumetric measurements from magnetic resonance imaging (MRI) in older adults [10, 11]. The literature indicates that higher physical activity levels, especially MVPA, are associated with a larger hippocampal volume and attenuated brain atrophy in older adults. By comparison, very little is known about the spectrum of physical activity levels, including MVPA, light physical activity, and sedentary behavior, relative to volumes of whole brain GM and WM and deep GM structures using MRI in persons with MS. To our knowledge, one study has demonstrated that physical activity, based on a nonspecific measure of movement counts per day, was associated with resting state functional connectivity between the hippocampus and posteromedial cortex (PMC) in 45 persons with MS [12].

The current study provides a novel examination of the association between objectively measured physical activity and MRI metrics of whole brain GM and WM and deep GM structures in people with MS. Based on previous research in older adults [10, 11], we hypothesized that those with MS who had higher levels of MVPA would have larger whole brain GM and WM volumes and hippocampal volume. We further examined the association between levels of physical activity and volumes of the thalamus and basal ganglia, as these deep GM structures are associated with disability and cognitive impairment in MS. We hypothesized that persons with MS who had higher levels of MVPA would have larger volumes of the thalamus and basal ganglia nuclei.

## 2. Methods

### 2.1. Participants

The methods for this study were approved by an institutional review board (IRB) and all participants provided written informed consent before enrollment. We enrolled 39 patients with clinically definite MS [13] who were recruited through targeted advertisements dispersed in central Illinois. The inclusion criteria were diagnosis of MS based on physician verification using accepted criteria [13], being relapse-free over the past 30 days, age between 18 and 64 years, ability to walk with or without an assistive device, willingness and ability to complete in-person assessments, minimal risk for engaging in physical activity (i.e., reported “yes” to less than two questions on the Physical Activity Readiness Questionnaire (PAR-Q) [14]), and physician approval. We excluded all persons with contraindications for MRI. This yielded a sample that was primary female (77%) with a mean age of 48.7 (SD = 9.6) years. The clinical course was relapsing-remitting MS (RRMS) in 30 patients (77%) and primary or secondary progressive MS in 9 patients (23%). The average disease duration was 10.3 (SD = 8.5) years. The median Expanded Disability Status Scale (EDSS) score was 4.5 (IQR = 2.5).

### 2.2. Physical Activity

Physical activity was objectively measured with ActiGraph model GT3X+ accelerometers (Health One Technology, Fort Walton Beach, FL) with data processed by researchers uninvolved in MRI acquisition and processing. The ActiGraph model GT3X+ contains a solid state, digital accelerometer that generates an electrical signal proportional to the force acting on it along three axes. The acceleration signal is sampled by a 12-bit analog-to-digital converter and stored in a raw format in the units of gravity (G's) with motion outside normal human movements rejected by a band-pass filter. The accelerometers were initially calibrated by the manufacturer before the onset of this study, and we further calibrated the accelerometers before and after the study such that there was less than 10% variation in output across a 15-minute period of walking at 1.34 meters/second on a motor-driven treadmill. The accelerometers were initialized using the low-frequency extension feature as this increases the sensitivity for capturing low-frequency accelerations (i.e., slow walking). The raw activity data were downloaded using software (ActiLife 6) and expressed as activity counts/minute. The data were processed into two separate Microsoft Excel files representing daily accelerometer wear time and time spent in sedentary behavior (i.e., ≤100 counts/minute), light physical activity (i.e., 100–1722 counts/minute), and MVPA (i.e., ≥1,723 counts/minute), based on activity count cut-points developed for persons with MS [15]. Accelerometer wear time data were checked against participant recorded wear times from the log sheet and only valid days (≥10 hours of wear time without periods of continuous zeros exceeding 60 minutes indicative of noncompliance) were included in the analysis.

### 2.3. MRI Acquisition and Analysis of DGM Structures

The MRI acquisition and analysis were conducted by personnel who were not involved in the assessment of physical activity. Using a whole body Siemens Trio 3 Tesla MRI scanner (Erlangen, Germany), we acquired high-resolution 3D T1-weighted structural brain images using an MPRAGE sequence with the following parameters: 23 cm FOV, 256 × 256 × 192 matrix size with 0.9 mm isotropic resolution, echo time (TE)/repetition time (TR)/inversion time (TI) of 2.32/1900/900 ms, flip angle of 9°, and GRAPPA accelerated factor of 2 with 24 reference lines. We then extracted the brain and excluded the skull by deleting nonbrain tissue from our T1 whole brain images using the Brain Extraction Tool [16] from FMRIB's Software Library (FSL). We used bias-field correction in order to ensure accurate brain extraction. We segmented the 3D T1 image into 3 tissue types, WM, GM, and cerebrospinal fluid (CSF), using FMRIB's Automated Segmentation Tool (FAST) [17] from FSL. Using the 3D T1 images, we calculated volumes of the right and left hippocampi, thalami, and basal ganglia nuclei using FMRIB's Integrated Registration and Segmentation Tool (FIRST) [18]. This segments the T1 images into subcortical structures and measures the volumes. We computed a volume scaling factor to account for differences in total intracranial volume by linear registration of the skull and brain masks using FSL's Linear Image Registration Tool (FLIRT) and extracting scaling parameters from the resulting affine transformation matrix, as is done in FSL's SIENAX [19–21]. We further computed whole brain WM and GM volumes using SIENAX. We then normalized all volumes based on intracranial volume by multiplication with this volume scaling factor, and then normalized right and left volumes were averaged for inclusion as composites in the statistical analyses. This was based on strong correlations between right and left volumes (e.g., thalami: *r* = .90, *p* < 0.0001) and reducing possible inflated error rates with multiple comparisons. We note that this process has been undertaken in other research on deep GM volumes in MS [22].

### 2.4. Procedures

All participants who qualified based on inclusion/exclusion criteria completed a demographic/clinical questionnaire, wore an accelerometer for a 7-day period, and underwent an MRI. The MRI occurred after collecting the 7 days of accelerometer data. The participants further underwent a neurological evaluation by a Neurostatus certified examiner for generation of an EDSS score. This was level C certification and performed by clinical laboratory staff without supervision of a neurologist.

### 2.5. Data Analysis

The data were analyzed in SPSS Statistics version 22.0 for Mac OS X operating system. Descriptive statistics were provided as mean (standard deviation) and range of scores. We examined the associations among physical activity and normalized whole brain GM volume (NGMV) and WM volume (NWMV) and hippocampal, thalamic, caudate, putamen, and pallidum scaled volumes using Pearson product-moment correlation (*r*) and then partial correlation (*pr*) coefficients. We opted for partial correlations as this analysis describes the strength of association between two variables when other variables are fixed. This is a simple approach for estimating the independent association between two variables considering a set of other correlated parameters. Correlations between variables were interpreted by guidelines of 0.1, 0.3, and 0.5 as small, moderate, and large, respectively [23]. The partial correlations controlled for days of valid accelerometer data and accelerometer wear time (min/day) or sex (0 = female, 1 = male), age (years), clinical course of MS (0 = RRMS, 1 = progressive MS), and EDSS score as covariates.

## 3. Results

### 3.1. Descriptive Statistics

The descriptive statistics for accelerometry (e.g., days of valid accelerometer data, accelerometer wear time (min)), physical activity (e.g., sedentary behavior (min/day), light physical activity (min/day), and MVPA behavior (min/day)), and scaled composite volumes (mm^3^) of normalized brain volume measures (e.g., NGMV and NWMV and hippocampus, thalamus, caudate, pallidum, and putamen) are provided in Table 1.

### 3.2. Correlations and Partial Correlations in MS

The correlations between levels of physical activity and scaled composite NGMV and NWMV and normalized deep GM volumes (mm^3^) are provided in Table 2. The bivariate correlations indicated significant associations between MVPA (min/day), but not light physical activity behavior (min/day) or sedentary behavior (min/day), and NWMV (*r* = .418, *p* < 0.01), hippocampus (*r* = .325, *p* < 0.05), thalamus (*r* = .404, *p* < 0.05), caudate (*r* = .418, *p* < 0.05), putamen (*r* = .341, *p* < 0.05), and pallidum (*r* = .454, *p* < 0.01) volumes (Model 1). The correlations remained significant between MVPA (min/day) and NWMV (*pr* = .359, *p* < 0.05), thalamus (*pr* = .352, *p* < 0.05), caudate (*pr* = .405, *p* < 0.01), and pallidum (*pr* = .407, *p* < 0.01), when controlling for days of valid accelerometer data and accelerometer wear time (min) (Model 2). The correlations further remained significant for the association of MVPA (min/day) and all brain volumetric measurements, including NGMV (*pr* = .370, *p* < 0.05), when controlling for sex, age, clinical course of MS, and EDSS (Model 3).

## 4. Discussion

This study examined the associations between objectively measured physical activity and MRI metrics of brain volume in persons with MS. The primary result was a consistent association between MVPA (min/day), but not light physical activity or sedentary behavior, and whole brain GM, whole brain WM, and deep GM structures including hippocampus, thalamus, caudate, putamen, and pallidum volumes. Persons with MS who had higher levels of MPVA (min/day) had larger volumes of whole brain GM and WM, hippocampus, thalamus, caudate, putamen, and pallidum. After controlling for sex, age, clinical course of MS, and EDSS as covariates, all brain volumetric measurements (e.g., whole brain GM, whole brain WM, hippocampus, thalamus, caudate, putamen, and pallidum) were significantly associated with MVPA (min/day).

Our results regarding MVPA, whole brain GM and WM, and hippocampal volumes are consistent with previous research in older adults [10, 11]. These studies report that higher levels of MVPA (min/day), but not light physical activity or sedentary behavior, were positively associated with attenuated whole brain GM and WM and hippocampal volumes. We provide the first evidence of similar associations between MVPA and other deep GM structures (e.g., thalamus, caudate, putamen, and pallidum) in persons with MS.

We recognize that directionality cannot be inferred from these results, as we conducted a cross-sectional assessment of physical activity and brain volume in persons with MS. MVPA could influence the volume of brain structures, or conversely, the integrity of brain structures might influence one's capacity for engaging in MVPA through cognitive or motor functions. However, we demonstrate nonuniform associations between levels of physical activity and brain volumes, such that only MVPA was significantly associated with changes in brain volumes. This leads us to believe that MVPA may be influencing the volume of brain structures. Indeed, a previous study demonstrated one year of moderate intensity aerobic exercise increased the size of the anterior hippocampus in 120 older adults [24]. Other studies indicate physical activity early in life may predict GM volume in late adulthood [25, 26]. Experimental and epidemiological research is warranted to identify if participation in MVPA influences brain volume among persons with MS.

Subsequent research may extend on this study by identifying a possible mechanism for the effect of MVPA on changes in brain volume in persons with MS. These possible mechanisms might include increases in neuronal viability and brain-derived neurotrophic factor (BDNF). For example, neurogenesis within the dentate gyrus, a subregion of the hippocampus, was stimulated by exercise in mice [27]. In animal models of Alzheimer's disease, the expression of growth factors, such as BDNF, reinstated hippocampal function following physical activity by promoting neurogenesis, angiogenesis, and synaptic plasticity [28]. An aerobic exercise training study in older adults demonstrated serum BDNF levels to be associated with changes in hippocampal volume, suggesting a possible link between physical activity and increased brain volume [22, 24]. Similar studies in persons with MS, as well as in the animal model of MS, experimental autoimmune encephalomyelitis (EAE), are warranted. Future research might examine the associations between brain volumes and MVPA with functional outcomes, such as walking and cognition dysfunction.

This study had many strengths but is not without important limitations. We did not assess other MRI variables such as diffusion tensor imaging, MR spectroscopy, and magnetization transfer or quantify regions of the motor cortex. We further did not conduct MRI of the spinal cord, and such data may be relevant especially in terms of mobility. We further did not include a healthy control group for comparative values and comparison of correlation coefficients. We controlled for days of valid accelerometer data, accelerometer wear time (min), sex, age, clinical course of MS, and EDSS, but there may be other variables that could confound the examination of associations between physical activity and brain volume. Another limitation is the lack of control for fatigue as this can be associated with gray matter atrophy [29] and physical activity [30].

## 5. Conclusion

Overall, this study provides novel evidence for MVPA as a correlate of whole brain GM and WM and deep GM matter structures in persons with MS. This result supports the possibility that enhancing physical activity, specifically MVPA, could contribute to brain health in people with MS. This is important considering brain volume is correlated with disability status and cognitive impairment in MS [7]. Further studies and behavioral interventions are warranted to confirm the benefits of physical activity on brain volume in people with MS.

## Figures and Tables

**Table 1:** Descriptive statistics for accelerometry, levels of physical activity, and scaled composite volumes of normalized brain volume measures in 39 persons with MS.

Variable	Mean (standard deviation)	Range
Days of valid accelerometer data (days)	6.1 (0.9)	4.0–7.0
Accelerometer wear time (min/day)	805 (78)	681–1010
Sedentary behavior (min/day)	594 (123)	407–1048
Light physical activity (min/day)	213 (83)	60–427
MVPA (min/day)	11.9 (15.4)	0.3–53.8
NGMV (mm^3^)	670873 (55423)	528164–784446
NWMV (mm^3^)	725122 (62592)	603643–848542
Hippocampus (mm^3^)	4214 (864)	2277–5591
Thalamus (mm^3^)	8571 (1222)	6265–10712
Caudate (mm^3^)	4020 (740)	2235–5349
Pallidum (mm^3^)	1868 (265)	1237–2261
Putamen (mm^3^)	5208 (905)	3209–6465

Note: MVPA = moderate-to-vigorous physical activity; NGMV = normalized gray matter volume; NWMV = normalized white matter volume.

**Table 2:** Correlations between levels of physical activity and scaled composite volumes of normalized brain volume measures in 39 persons with MS.

Variable	Model 1			Model 2			Model 3		
	MVPA (min/day)	Light physical activity (min/day)	Sedentary behavior (min/day)	MVPA (min/day)	Light physical activity (min/day)	Sedentary behavior (min/day)	MVPA (min/day)	Light physical activity (min/day)	Sedentary behavior (min/day)
NGMV (mm^3^)	.304	.215	−.099	.297	.167	−.156	.370^*∗*^	.219	.059
NWMV (mm^3^)	.418^*∗∗*^	.235	.124	.359^*∗*^	.126	−.029	.433^*∗∗*^	.171	.281
Hippocampus (mm^3^)	.325^*∗*^	.140	−.046	.284	.065	−.151	.499^*∗∗*^	.018	.164
Thalamus (mm^3^)	.404^*∗*^	.163	.078	.352^*∗*^	.065	−.058	.380^*∗*^	.024	.228
Caudate (mm^3^)	.418^*∗*^	.267	−.065	.405^*∗∗*^	.227	−.123	.539^*∗∗*^	.198	.099
Putamen (mm^3^)	.341^*∗*^	.218	.015	.303	.144	−.072	.369^*∗*^	.168	.125
Pallidum (mm^3^)	.454^*∗∗*^	.198	.162	.407^*∗∗*^	.074	.027	.498^*∗∗*^	.145	.253

Note: Model 1 = Pearson product-moment correlations (*r*); Model 2 = partial Pearson product-moment correlations (*pr*), controlling for days of valid accelerometer data and accelerometer wear time; Model 3 = partial Pearson product-moment correlations (*pr*), controlling for sex, age, clinical course of MS, and EDSS; ^*∗∗*^correlation is significant at the 0.01 level (2-tailed); ^*∗*^correlation is significant at the 0.05 level (2-tailed); MS = multiple sclerosis; MVPA = moderate-to-vigorous physical activity; NGMV = normalized gray matter volume; NWMV = normalized white matter volume.
